# Disulfiram overcomes bortezomib and cytarabine resistance in Down-syndrome-associated acute myeloid leukemia cells

**DOI:** 10.1186/s13046-017-0493-5

**Published:** 2017-02-01

**Authors:** Ranjan Bista, David W. Lee, Oliver B. Pepper, David O. Azorsa, Robert J. Arceci, Eiman Aleem

**Affiliations:** 1 Institute of Molecular Medicine at Phoenix Children’s Hospital, Phoenix, AZ USA; 2 Department of Child Health, University of Arizona College of Medicine-Phoenix, Biosciences Partnership Building (BSPB), 5th floor, 475 N 5th Street, Phoenix, AZ 85004 USA; 3 Department of Biology and Biochemistry, University of Bath, Bath, UK; 4 Department of Zoology, Faculty of Science, Alexandria University, Alexandria, Egypt

**Keywords:** Relapsed acute myeloid leukemia, Down syndrome-associated AML, Chemoresistance, Disulfiram, Bortezomib, Cytarabine, ALDH

## Abstract

**Background:**

Children with Down syndrome (DS) have increased risk for developing AML (DS-AMKL), and they usually experience severe therapy-related toxicities compared to non DS-AMKL. Refractory/relapsed disease has very poor outcome, and patients would benefit from novel, less toxic, therapeutic strategies that overcome resistance. Relapse/resistance are linked to cancer stem cells with high aldehyde dehydrogenase (ALDH) activity. The purpose of the present work was to study less toxic alternative therapeutic agents for relapsed/refractory DS-AMKL.

**Methods:**

Fourteen AML cell lines including the DS-AMKL CMY and CMK from relapsed/refractory AML were used. Cytarabine (Ara-C), bortezomib (BTZ), disulfiram/copper (DSF/Cu^2+^) were evaluated for cytotoxicity, depletion of ALDH-positive cells, and resistance. BTZ-resistant CMY and CMK variants were generated by continuous BTZ treatment. Cell viability was assessed using CellTiter-Glo®, ALDH activity by ALDELUOR^TM^, and proteasome inhibition by western blot of ubiquitinated proteins and the Proteasome-Glo™ Chymotrypsin-Like (CT-like) assay, apoptosis by Annexin V Fluos/Propidium iodide staining, and mutations were detected using PCR, cloning and sequencing.

**Results:**

Ara-C-resistant AML cell lines were sensitive to BTZ and DSF/Cu^2+^. The Ara-C-resistant DS-AMKL CMY cells had a high percentage of ALDH^bright^ “stem-like” populations that may underlie Ara-C resistance. One percent of these cells were still resistant to BTZ but sensitive to DSF/Cu^2+^. To understand the mechanism of BTZ resistance, BTZ resistant (CMY-BR) and (CMK-BR) were generated. A novel mutation *PSMB5* Q62P underlied BTZ resistance, and was associated with an overexpression of the β5 proteasome subunit. BTZ-resistance conferred increased resistance to Ara-C due to G1 arrest in the CMY-BR cells, which protected the cells from S-phase damage by Ara-C. CMY-BR and CMK-BR cells were cross-resistant to CFZ and MG-132 but sensitive to DSF/Cu^2+^. In this setting, DSF/Cu^2+^ induced apoptosis and proteasome inhibition independent of CT-like activity inhibition.

**Conclusions:**

We provide evidence that DSF/Cu^2+^ overcomes Ara-C and BTZ resistance in cell lines from DS-AMKL patients. A novel mutation underlying BTZ resistance was detected that may identify BTZ-resistant patients, who may not benefit from treatment with CFZ or Ara-C, but may be responsive to DSF/Cu^2+^. Our findings support the clinical development of DSF/Cu^2+^ as a less toxic efficacious treatment approach in patients with relapsed/refractory DS-AMKL.

**Electronic supplementary material:**

The online version of this article (doi:10.1186/s13046-017-0493-5) contains supplementary material, which is available to authorized users.

## Background

Although the clinical outcome of pediatric acute myeloid leukemia (AML) has improved over the past few decades, approximately 30% of patients relapse, and outcome is poor with about 30–40% survival [1]. The remission induction therapy for AML consists of 4–5 cycles of intensive chemotherapy, which typically includes cytarabine (Ara-C), the backbone for many therapeutic regimens, combined with etoposide and anthracycline [2]. Acquired resistance to Ara-C is a major obstacle in the clinical management of AML. Increasing the intensity of the current chemotherapy regimens does not improve outcomes because of the high percentage of treatment-related deaths (5–10%), and of long-term side effects [3].

Children with Down syndrome have a substantially increased risk for developing acute leukemia (10–30 fold) [4]. They present with a unique subtype; acute megakaryoblastic leukemia, usually following a transient myeloproliferative disorder in the neonatal period that is characterized by somatic mutations in the *GATA1* gene [2]. Patients with Down syndrome-associated AML (DS-AMKL) have increased toxicities after treatment with chemotherapy compared to non-DS children with AML, which prevents the use of higher chemotherapy doses. For those patients who then relapse, they have poorer outcome [5]. It was reported that following stem cell transplants, patients with DS-AMKL had an overall survival (OS) of 19% [6]. It is concluded from these studies that DS patients with refractory/relapsed AML have extremely chemotherapy-resistant disease [7], which urgently requires novel therapeutic strategies that can overcome relapse and resistance with the least toxicity. There is evidence linking disease relapse and chemotherapy resistance to cancer stem cells with high aldehyde dehydrogenase (ALDH) activity [8]. One agent that appears to deplete the AML stem cell population, and to act synergistically with conventional chemotherapy agents, is the proteasome inhibitor bortezomib (Velcade) (BTZ) [9], which has been recently introduced in clinical trials for the treatment of AML [10]. BTZ reversibly inhibits the chymotrypsin-like activity (CT-like) at the β5-subunit (PSMB5) of the 26S proteasome. The CT-like activity is associated with the rate-limiting step of proteolysis [11]. BTZ was previously shown to induce apoptosis in ALL and AML cell lines [9, 12, 13] and in nude mice xenografts [14–16]. Currently, there are three clinical trials evaluating BTZ in children with relapsed/refractory AML (NCT02419755, NCT02551718, NCT01950611). However, acquired BTZ resistance and its toxicity limit its efficacy [17]. The mechanisms of BTZ resistance include, but are not limited to mutations of *PSMB5* and the up-regulation of proteasome subunits [18].

An attractive strategy receiving increasing interest in cancer therapeutics is re-purposing drugs that have previously been approved by the FDA for other indications. One such drug is Disulfiram (DSF), which has been used clinically for the last 60 years for the treatment of alcoholism. DSF functions by irreversibly inhibiting ALDH [19]. DSF has been shown to have in vitro and in vivo anticancer properties against various types of cancers [20–26]. DSF is also used in clinical trials for adult glioblastoma, melanoma, prostate, pancreatic, and liver cancers (clinicaltrials.gov). The antineoplastic activity of DSF, that is copper-dependent [21–23], has been principally attributed to proteasome inhibition [25, 27], generation of reactive oxygen species [20, 23], and inhibition of methylguanine-DNA-methyltransferase [26]. DSF is an oral drug, very well tolerated in adult patients, and inexpensive, which makes it an attractive candidate for consideration in the treatment of pediatric DS-AMKL. The purpose of the present work was to study less toxic alternative therapeutic agents for pediatric relapsed/refractory DS-AMKL using an in vitro approach. Using the Ara-C-resistant DS-AMKL cell line, CMY, we found a small percentage of cells that also showed resistance to BTZ. However, this small population of dual drug resistant cells was sensitive to DSF/Cu^2+^. In order to determine the mechanism of resistance, we developed an in vitro model of BTZ resistance in both CMY, and the Ara-C-sensitive DS-AMKL CMK cell lines, and found that DSF/Cu^2+^ could overcome BTZ resistance in this experimental setting.

## Methods

### Cell culture

Human AML cell lines (Table 1) were obtained from the American Type Culture Collection (ATCC) except for the M-07e, Molm-13 and NB4 cell lines that were available, and previously used, in our lab. CMY, CMK and CMS cell lines were gifts from Dr. Jeffrey W. Taub, Wayne State University, USA. All cell lines were authenticated at the University of Arizona Genetics Core (UAGC) using Short Tandem Repeat (STR) analysis. CMY and CMK are derived from Down syndrome patients with acute megakaryoblastic leukemia (AMKL), while CMS and M-07e are derived from Non-DS-AMKL. AML-193 (acute monocytic leukemia), THP-1 (MLL-AF9 rearranged AML), Molm-13 and MV-4-11 are from acute monocytic leukemia with Flt-3 ITD mutation, Kasumi-1 is from AML with t (8,21). TF-1 and HEL 92.1.7 are from erythroleukemia, while KG-1a is from an undifferentiated AML clone derived from KG-1 (erythroleukemia). HL60 and NB4 are from acute promyelocytic leukemia.

**Table 1:** Cytotoxicity of BTZ, Ara-C and DSF/Cu^2+^ in AML cell lines

Cell lines	AML Classification	Age	Type	IC_50_ (nM) (Mean ± SD)		
				BTZ	Ara-C	DSF/Cu^2+^
CMK	Acute Megakaryoblastic Leukemia	10 months	Down Syndrome Relapse	4.9 ± 2.6	70 ± 30	105 ± 25
CMY	Acute Megakaryoblastic Leukemia	21 months	Down SyndromeRefractory	2.6 ± 0.6	1795 ± 391	72 ± 16
CMS	Acute Megakaryoblastic Leukemia	2 years		4.3 ± 1.6	154 ± 21	82 ± 4.7
THP-1	Acute Monocytic Leukemia MLL-AF9	1 year	Relapse	7.8 ± 3.3	3537 ± 331	79 ± 18
M-07e	Acute Megakaryoblastic Leukemia	1 year		3.7 ± 0.8	24 ± 4.4	68 ± 22
AML-193	Acute Monocytic Leukemia	13 years	Relapse	4.6 ± 3.1	414 ± 29.9	53 ± 12.7
Kasumi-1	AML t(8,21)	7 years	2nd relapse after BMT	6.7 ± 3.0	27.6 ± 2.5	89 ± 13
Molm-13	AML with Flt-3 ITD	20 years	Relapse	6.8 ± 3.6	4 ± 1	98 ± 10
MV-4-11	Biphenotypic Myelomonocytic Leukemia FLT-3 ITD	10 years		3.4 ± 0.5	278 ± 73	50 ± 10
NB4	Acute Promyelocytic Leukemia	20 years	Relapse	2.7 ± 1.4	123 ± 114	57 ± 5
HL-60	Acute Promyelocytic Leukemia	36 years		7.1 ± 1.5	424 ± 172	67 ± 19
KG-1a	Acute Myeloid leukemia	59 years		17.0 ± 0.9	177 ± 43	79 ± 11
TF-1	Erythroleukemia	35 years		8.3 ± 2.1	75 ± 52	101 ± 21
HEL 92.1.7	Erythroleukemia	30 years		6.2 ± 2.9	102 ± 13	100 ± 8.5

IC_50_ values are presented as mean ± standard deviation. Data represent at least three replicate experiments. *Ara-C* cytarabine, *BTZ* bortezomib, *DSF* disulfiram

CMY, CMK, CMS, THP-1, NB-4, Kasumi-1, Molm-13, M-07e, TF-1 and HEL 92.1.7 cell lines were routinely cultured in Roswell Park Memorial Institute 1640 medium (RPMI 1640) supplemented with 10% fetal bovine serum (FBS; ATLAS Biologicals, Ft. Collins, CO). M-07e and TF-1 cells were supplemented with 10 ng/ml and 2 ng/ml granulocyte macrophage colony-stimulating factor (GM-CSF), respectively. HL-60, KG1a, MV-4-11, and AML-193 cells were routinely cultured in Iscove’s modified Dulbecco’s medium (IMDM) supplemented with 20% FBS for HL-60 and KG-1a, 10% FBS for MV-4-11, and 5% FBS, 5 ng/ml GM-CSF and 5 μg/ml insulin for AML-193. HEK 293A, embryonic kidney cells (Invitrogen/LifeTech, Carlsbad, CA) were cultured in Dulbecco’s modified Eagle medium (DMEM) containing 10% FBS. All growth media were supplemented with 2 mM l-glutamine, 100 units/mL penicillin, and 100 g/mL streptomycin, and cells were maintained at 37 °C in a humidified incubator with 5% CO_2_. All media, antibiotics, and l-Glutamine were purchased from Invitrogen/LifeTech. All growth factors were purchased from R & D Systems (Minneapolis, MN). To generate bortezomib-resistant (BR) AML cell lines, CMY and CMK cells were exposed to stepwise increasing concentrations of BTZ (Selleck Chemicals Houston, TX,) over a period of 9 months, starting at a concentration of 5 nM (IC_90_ dose) up to a concentration of 200 nM in CMY, and 100 nM in CMK cells. Disulfiram, Copper (II) D-gluconate, dimethylsulfoxide (DMSO) and MG-132 were purchased from Sigma-Aldrich (St. Louis, MO). Cytarabine (Ara-C), Carfilzomib (CFZ), Etoposide (VP-16) and Daunorubicin were purchased from Selleck Chemicals. All compounds were resuspended in DMSO and stored in −80 °C until use.

### Cell viability assay

AML cells were seeded in 4 replicates in 384 well plates (Greiner Bio One, Monroe, NC) at a density of 3000 cells/well in 40 μL. Cells were incubated overnight at 37 °C, 5% CO_2_ then treated with serial dilutions of freshly prepared Ara-C, BTZ, DSF/Cu^2+^, MG132, CFZ, VP-16 or daunorubicin, in a 10 μL volume. Cell viability was assessed after 72 h drug exposure using CellTiter-Glo® Luminescent Cell Viability Assay (Promega, Madison, WI) and plates were read using the EnVision plate reader (Perkin Elmer, Waltham, MA). IC_50_ values were calculated, and drug dose response curves (DDR) were prepared using Prism version 6.0 d (GraphPad Software, Inc., La Jolla, CA).

### Aldefluor assay and cell sorting

The ALDH activity was measured using a fluorogenic dye-based assay; ALDELUOR™ kit (Stem Cell Technologies, Seattle, WA) according to manufacturer’s instructions followed by flow cytometry. Cells were incubated in Aldefluor assay buffer containing an ALDH substrate, bodipy-aminoacetaldehyde (1 μmol/l per 1x10^6 cells), for 30 min at 37 °C. As a negative control, a fraction of the cells from each sample was incubated under identical conditions in the presence of the ALDH inhibitor diethyl-aminobenzaldehyde (DEAB). The FACSAria II flow cytometer (BD Biosciences, San Diego, CA, USA) was used to assess and sort the ALDH^bright^ and the ALDH^low^ cell populations. The data were analyzed using FACS DIVA software (BD Biosciences).

### Analysis of cell cycle and apoptosis

For cell cycle analysis, CMY and CMY-BR cells were plated at (5x10^5^ cells) per 100 mm culture dish and allowed to grow for 24 h, then treated with the indicated concentrations of Ara-C. Cell cycle phase distribution was analyzed by flow cytometry using propidium iodide (PI) as described in [28]. For apoptosis analysis, cells were treated with various concentrations of DSF/Cu^2+^ for 24 h and apoptosis was assessed using the Annexin V-FLUOS kit (Roche, Indianapolis, IN) and PI as described in [28]. The samples were analyzed with FACSAria II (BD Biosciences), and the data were analyzed using the FACS DIVA software (BD Biosciences).

### Western blot analysis

AML cells were seeded in 6-well plates at 5x10^5^ cells per well, and incubated overnight at 37 °C and 5% CO_2_. Drug-treated AML cells were lysed in modified RIPA buffer (50 mM Tris pH 7.4, 150 mM NaCl, 1% NP-40, 1 mM EDTA, 0.25% sodium deoxycholate) containing Halt™ protease inhibitor and phosphatase inhibitor cocktail (Thermo Scientific, Pittsburgh, PA) on ice for 20 min. Lysates were centrifuged at 4 °C at 13000 x *g* for 20 min and supernatants stored at −80 °C. Protein concentrations were determined using the Pierce™ BCA Protein Assay (Thermo Fisher). Equal amounts of protein were resolved on 4-12% SDS-PAGE MiniPROTEAN® precast gradient gels (Bio-Rad Laboratories Inc., Hercules, CA) and transferred to Immun-Blot® LF polyvinylidene difluoride (PVDF) membranes (Bio-Rad). Membranes were blocked with 5% BSA in Tris buffered saline containing 0.1% Tween® 20 (v/v), incubated overnight at 4 °C with mouse or rabbit antibodies against ubiquitin, Poly ADP Ribose Polymerase (PARP) (Cell Signaling Technology, Danvers, MA) and the 20S β5 proteasome subunit (Enzo Life Science, Farmingdale, NY), or against tubulin or GAPDH (Santa Cruz Biotechnology, Dallas, TX) as loading control. Membranes were washed, incubated with horseradish peroxidase (HRP)-conjugated anti-rabbit or anti-mouse secondary antibodies (SCBT), followed by exposure to Immobilon™ Western Chemiluminescent HRP Substrate (Millipore, Billerica, MA, USA). Membrane blots were exposed using the ChemiDoc™ MP Imaging System (Bio-Rad).

### Assessment of chymotrypsin-like activity

Cells were seeded in a 364 well plate at 5,000 cells in 15 μL of medium/well in 4 replicates and treated with DSF/Cu^2+^or BTZ and incubated for the indicated time. The CT-Like activity was measured using the Proteasome-Glo™ Chymotrypsin-Like cell based assay (Promega) and the plate was read using the SpectraMax® Paradigm plate reader (Molecular Devices, Sunnyvale, CA).

### Cloning, DNA sequencing and transfection

To clone the *PSMB5* subunit, RNA was isolated from CMY-BR200 and from the parental CMY cell lines using RNeasy mini kit (Qiagen, Gaithersburg, MD). cDNA was prepared by reverse transcription using iScript™ (Bio-Rad) and whole length *PSMB5* was amplified by PCR using the following primer set: Forward: 5′-ATTAGCTAGCAGACATGGCGCTTGCCAGCGTGTT-3′ containing the NheI restriction site (GCTAGC), and Reverse: 5′-TATACTCGAGTCAGGGGGTAGAGCCACTATACTTCT-3′ containing the XhoI restriction site (CTCGAG) (LifeTechnologies). The full length *PSMB5* from CMY (WT) and CMY-BR200 underwent PCR purification clean up (Qiagen). PCR products, and pcDNA 3.1 Hygro (+) plasmid vector (LifeTechnologies) were then each treated with NheI and XhoI restriction enzymes (NEB, Ipswich, MA, USA) for preparation for cloning. Digested products were separated on an agarose gel, extracted and purified (Qiagen). Full-length PCR products were ligated into plasmid vector and transformed into *E. coli* One Shot Top 10, (Life Technologies) for cloning (ampicillin selection). Positive colonies were expanded and plasmid DNA was purified (Qiagen Midiprep kit). DNA sequencing was performed at the UAGC core facility. HEK 293A cells were grown in 6-well plates and transfected with 4.2 μg of plasmid DNA using X-tremeGENE HP (Roche Diagnostics). Transfected cells were selected after two days using hygromycin 200 μg/mL. Sequence alignment was done using SnapGene (GSL Biotech LLC, Chicago, IL).

### Quantitative real time RT-PCR

RNA and cDNA from CMY and CMY-BR cells were prepared as described above. Quantitative real time RT-PCR was performed using (BioRad CFX96) thermocycler using 1 μL/replicate of cDNA, 12.5 μL of SYBR green (BioRad), in a total of 25 μL reaction volume in triplicate. The following primers for real-time amplification of *PSMB5* were used: Forward 5′-TGTAGCAGCTGCCTCCAAAC-3′; reverse: 5′-AGGTGGCCCCTGAAATCCGG-3′ (Invitrogen/LifeTech). GAPDH (F:5′-TGGACCTGACCTGCCGTCTA-3′; R: 5′-AGGAGTGGGTGTCGCTGTTG-3′) was used to normalize the mRNA expression from CMY. The mRNA expression was analyzed using the 2^- **∆∆**Ct^ method and expressed as fold change.

### Colony formation assay

Cells were plated in 35-mm dishes in triplicates at low densities in Methocult H4100 (Stem Cell Technologies) and treated with different concentrations of drugs vs. no drug control. Cells were incubated for 7 to 12 days. The colonies were counted using a Nikon TMS microscope and compared with untreated cells.

### Statistical significance

Data are presented as means ± standard error. Statistical significance was determined by two-tailed paired *t*-test or by one-way analysis of variance for multiple comparisons using GraphPad Prism V. 6.0. *P* values of *≤ 0.05* were considered statistically significant.

## Results

### DS-AMKL cell lines with relative resistance to Ara-C are sensitive to BTZ and DSF/Cu^2+^

**Fig. 1:**
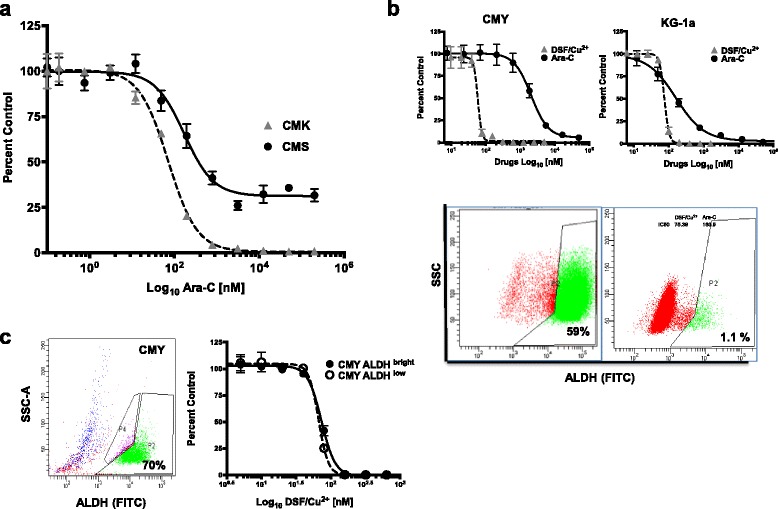
DSF/Cu^2+^ is cytotoxic in AML cell lines including Ara-C-resistant DS-AMKL cells independent of their endogenous ALDH activity. The DS-AMKL CMK and the non-DS-AMKL CMS cell lines were treated with different concentrations of Ara-C for 72 h and viability, expressed as percent of DMSO treated controls, was measured using CellTiter-Glo (CTG) assay. Drug dose response (DDR) curves show that DS-AMKL cell line CMK was more sensitive to Ara-C than the non DS-AMKL cell line CMS, with IC_50_ values of 71.16 nM and 170 nM, respectively (**a**). The DS-AMKL cell line CMY and the non-DS-AML cell line KG-1a were treated with different concentrations of Ara-C or DSF/Cu^2+^ for 72 h. CMY cells were relatively resistant to Ara-C (IC_50_ 1131 nM), but sensitive to DSF/Cu^2+^ (IC_50_ 83 nM). Although CMY cells had a 59% of ALDH^bright^ cell population, and KG-1a cells had a 1.1% ALDH^bright^ cell population (lower panel) they were both equally sensitive to DSF/Cu^2+^ (**b**). ALDH activity was measured using ALDELUOR^TM^ assay and flow cytometry. Dot plots in B show the percentage of ALDH-positive cells (FITC) on the x-axis, and the sideward scatter (SSC) on the y-axis. The gated cell populations were created using the ALDH inhibitor N, N-diethylaminobenzaldehyde (DEAB) provided with the kit. The ALDH^bright^ and the ALDH^low^ cell populations from the CMY cell line were flow-sorted and treated with different concentrations of DSF/Cu^2^ for 72 h and the viability was measured using CTG (**c**). Viability is presented as percentage viable cell relative to control (DMSO-treated) (y-axis) plotted against the Log_10_ nM of drug concentrations. Experiments were repeated at least three times

We used a panel of 14 AML cell lines including those developed from pediatric patients with relapsed/refractory DS-AMKL (CMY and CMK) to investigate their sensitivity and resistance to Ara-C (Table 1). The IC_50_ values for the AML cell lines treated with Ara-C ranged from 4 nM to 3.5 μM. While the CMK cell line was more sensitive to Ara-C (IC_50_ 70 ± 30 nM) in comparison to the non DS-AMKL CMS cell line (IC_50_ 154 ± 21 nM), CMY cells were 25-fold more resistant to Ara-C (IC_50_ 1795 ± 391 nM) in comparison to CMK cells (Table 1, Fig. 1a, b). As the refractory CMY cell line had a high percentage of the ALDH-positive “stem-like” cells (Fig. 1b), we investigated the cytotoxicity of BTZ (previously reported to target AML stem cells), and DSF (ALDH inhibitor) using the same panel that includes cell lines with high and low endogenous ALDH activity (Table 1, Fig. 1b). Since DSF cytotoxicity is copper-dependent, a constant concentration of 1 μM copper was used in the present study in combination with DSF (DSF/Cu^2+^) to mimic the physiological human free serum copper level. Both BTZ and DSF/Cu^2+^ were highly cytotoxic in all 14 AML cell lines including the relatively Ara-C-resistant cell lines CMY (Fig. 1b) and THP-1. The IC_50_ values of BTZ ranged from 2.6 nM in the DS-AMKL cell line CMY to 17 nM in KG1-a, while those of DSF/Cu^2+^ ranged from 50 to 105 nM (Table 1). These results indicated that DSF/Cu^2+^ and BTZ are highly cytotoxic in all AML cell lines including those with relative resistance to Ara-C.

### ALDH^bright^ cell populations resistant to BTZ are sensitive to DSF/Cu^2+^

**Fig. 2:**
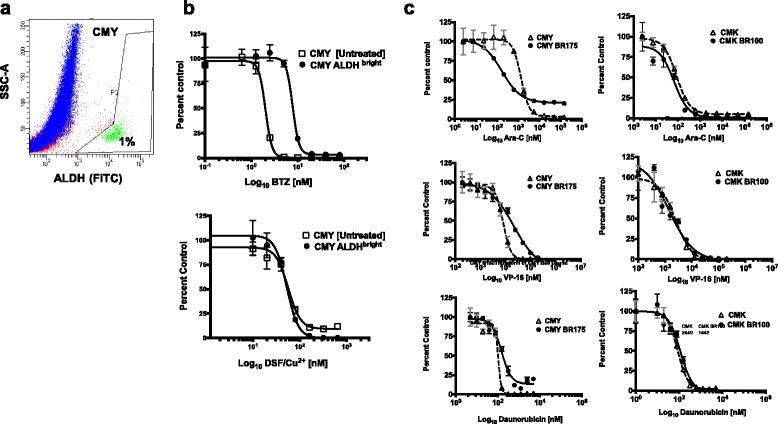
ALDH^Bright^ cells are resistant to BTZ but sensitive to DSF/Cu^2+^, and BTZ-resistance confers increased resistance to Ara-C in CMY cells. CMY cells were treated with 5 nM BTZ and processed for ALDH activity using ALDELUOR^TM^ assay and flow cytometry. Dot plot shows the percentage of ALDH-positive cells (FITC) on the x-axis, and the sideward scatter (SSC-A) on the y-axis. The gated cell populations were created using the ALDH inhibitor DEAB provided with the kit. One percent of the ALDH^Bright^ cell population was resistant to BTZ (**a**). Because these cells may play a role in BTZ resistance, this subpopulation was flow-sorted and subsequently subjected to drug dose response assays of either DSF/Cu^2+^ or a re-exposure to BTZ. The BTZ-resistant ALDH^bright^ subpopulation of CMY cells was still resistant to BTZ (IC_50_ 7.7 nM) compared to untreated, unsorted CMY cells (IC_50_ 2 nM), but were sensitive to DSF/Cu^2+^ (IC_50_ 56 nM vs 52 nM for untreated, unsorted CMY cells) (**b**). BTZ-resistant variants from CMY and CMK cell lines (CMY-BR and CMK-BR) were generated by exposure to stepwise increasing concentrations of BTZ up to 200 nM for CMY, and 100 nM for CMK. These cells were treated with different doses of Ara-C, VP-16 and daunorubicin. Dose response curves were plotted in comparison to the CMY and CMK parent cell lines. Approximately 25% of the CMY-BR cells remained viable after treatment with high Ara-C doses for 72 h, but the parental cells completely died at the same doses. The CMK cell line and its BTZ-resistant variant (CMK-BR) were both equally sensitive to Ara-C, VP-16 and daunorubicin (**c**)

Since DSF/Cu^2+^ is a potent, irreversible inhibitor of the stem-cell marker ALDH [23, 24, 29], we next studied whether DSF/Cu^2+^ is preferentially cytotoxic to AML cells with high endogenous ALDH activity. CMY and KG-1a cells (59% and 1.1% ALDH^bright^ cells, respectively), were treated with different concentrations of DSF/Cu^2+^. The two cell lines both showed sensitivity to DSF/Cu^2+^ indicating that the cytotoxicity of DSF/Cu^2+^ is not associated with the endogenous levels of ALDH in the cells (Table 1, Fig. 1b). Next, the ALDH^bright^ and the ALDH^low^ cell populations from the CMY cell line were flow-sorted and treated with different concentrations of DSF/Cu^2+^. The DSF/Cu^2+^ dose response curves were nearly identical for both cell populations with IC_50_ values of 73 nM for ALDH^bright^ and 66 nM for ALDH^low^ cells, indicating that both cell subpopulations were equally sensitive to DSF/Cu^2+^ (Fig. 1c). BTZ treatment (5 nM, 48 h) of the same CMY cells reveals a subpopulation (1%) of the ALDH^bright^ cells that are never depleted. (Fig. 2a). Because these cells may play a role in BTZ resistance, this subpopulation was flow-sorted and subsequently subjected to drug dose response assays of either DSF/Cu^2+^ or a re-exposure to BTZ, compared against untreated, un-sorted CMY cells as a control population (Fig. 2b). The BTZ-resistant ALDH^bright^ subpopulation of CMY cells showed increased resistance to BTZ compared to its control population. However, this same subpopulation of cells showed no change in sensitivity relative to the control population following treatment with DSF/Cu^2+^ (Fig. 2b). This confirmed the potential efficacy of DSF/Cu^2+^ in overcoming the residual BTZ-resistant fraction of cells in this experimental setting.

### BTZ-resistance in CMY cell line confers increased resistance to Ara-C compared to the parental counterpart

**Fig. 3:**
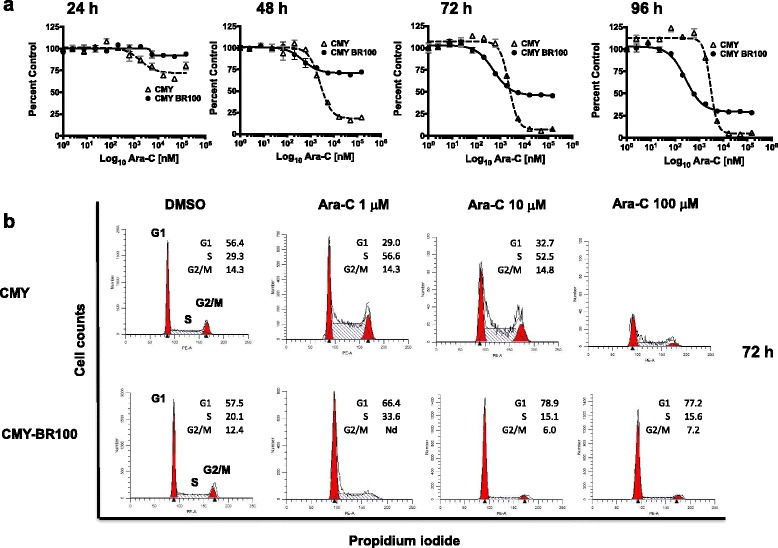
BTZ resistance protects CMY cells from S phase arrest by Ara-C. CMY and CMY-BR100 cell lines were treated with increasing concentrations of Ara-C, from 1 nM to 150 μM and viability plotted as percent of control (y-axis) against Log_10_ nM Ara-C (x-axis). After 72 h almost all the CMY parent cells died at high Ara-C doses, but the viability of CMY-BR cells remained at 50% at the same concentration and time points. After 4 days of exposure to high doses of Ara-C, the CMY-BR cells still maintained 25% viability (**a**). Cell cycle phase distribution of CMY and CMY-BR cell lines after treatment with 1, 10 and 100 μM Ara-C at 72 h were analyzed. CMY cells demonstrated an S-phase arrest at 1 and 10 μM Ara-C, with cell death at 100 μM. However, the CMY-BR cells showed an accumulation in the G1 phase, no S-phase arrest regardless of the Ara-C concentration used. (**b**). Cell cycle was analyzed using propidium iodide (PI) staining and flow cytometry. The plots show PI staining at the x-axis and cell counts at the y-axis. The graphs were prepared using ModFit LT™ software (Verity Software House). Nd: not detected

To study BTZ resistance in DS-AMKL, and its interaction with conventional chemotherapeutic agents used in the treatment of AML, we generated BTZ-resistant variants from the CMY and CMK cell lines, respectively, designated here as CMY-BR and CMK-BR, by exposure to stepwise increasing concentrations of BTZ up to 200 nM in CMY, and 100 nM in CMK (Table 2). Next we studied the cytotoxicity of Ara-C, VP-16 and daunorubicin in BTZ-resistant cell lines. Interestingly, while approximately 25% of the CMY-BR cells remained viable after treatment with high Ara-C concentrations for 3 days, the parental cells completely died at the same concentrations. Also, the IC_50_ values for VP-16 and daunorubicin each increased in the CMY-BR variant cell lines (Fig. 2c). Conversely, the CMK cell line and its BTZ-resistant variant (CMK-BR) were both equally sensitive to Ara-C, VP-16 and daunorubicin (Fig. 2c). It is known that Ara-C damages the DNA during S phase. In order to understand the mechanism by which BTZ resistance alters the response of CMY cells to Ara-C, we first studied the viability of CMY and CMY-BR over five days after treatment with increasing concentrations of Ara-C, from 1 nM to 150 μM (Fig. 3a). Interestingly, after 72 h almost all the CMY parent cells died after treatment with increasing concentrations of 16, 50 and 150 μM Ara-C, but the viability of CMY-BR cells remained at 50% at the same time points. After 4 days of exposure to these high concentrations of Ara-C, the CMY-BR cells still maintained 25% viability (Fig. 3a).

**Table 2:** IC_50_ of BTZ-resistant cells to DSF/Cu^2+^, CFZ and MG-132

	BTZ		CFZ		MG-132		DSF/Cu^2+^	
	IC_50_ (nM)	Fold	IC_50_ (nM)	Fold	IC_50_ (nM)	Fold	IC_50_ (nM)	Fold
CMY	2.9 ± 1.2		1.6 ± 0.4		210 ± 92		68 ± 14	
CMY BR100	175 ± 59	60.4	44.7 ± 2.6	25.2	2345 ± 192	11.2	82 ± 15	1.2
CMY BR200	206 ± 40	71.0	55.5 ± 8.4	31.4	2540 ± 380	12.1	97 ± 27	1.4
CMK	4.3 ± 0.6		4 ± 1.6		303 ± 63		115 ± 30	
CMK BR100	142 ± 46	33.0	73.7 ± 8	16.7	3512 ± 689	11.6	109 ± 15	0.95

IC_50_ values are presented as mean ± standard deviation and expressed as fold difference compared to parent cell line. Data represent at least three replicate experiments. *Ara-C* cytarabine, *BTZ* bortezomib, *CFZ* carfilzomib, *DSF* disulfiram

We next studied the cell cycle of both CMY and CMY-BR cell lines after treatment with 1, 10 and 100 μM Ara-C at 72 h (Fig. 3b). Upon treatment with Ara-C for 72 h, the CMY cells arrested in S-phase at 1 and 10 μM, and cells died at 100 μM. The CMY-BR cells accumulated in the G1 phase, but no similar S-phase arrest was observed even after treatment with 100 μM Ara-C at 72 h. These results indicated that BTZ-resistance might protect cells from DNA-damage by Ara-C.

### A novel mutation *PSMB5* Q62P underlies BTZ resistance in CMY cells

**Fig. 4:**
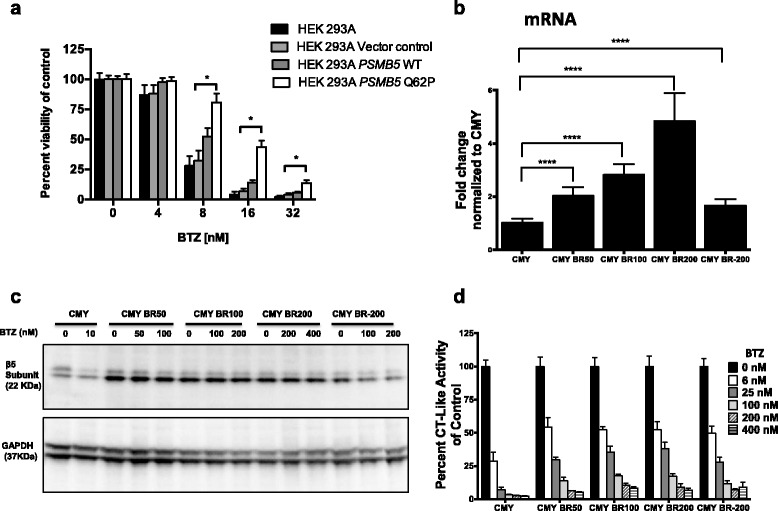
Transfection of *PSMB5* Q62P mutant gene confers BTZ resistance in HEK293A cell lines. The full length *PSMB5* WT and *PSMB5* Q62P mutant genes were amplified from CMY and CMY-BR cell lines, cloned in pcDNA 3.1 Hygro (+) and transfected into HEK293A cells. HEK293A cells (untreated), transfected with *PSMB5* WT, *PSMB5* Q62P, or empty vector were treated with several concentrations of BTZ, and viability was measured and presented as percentage viable cell relative to control (untreated) (y-axis) plotted against BTZ (nM) concentrations. HEK293A cells transfected with *PSMB5* Q62P showed increased resistance to BTZ compared to WT, vector only, and cells only, in a dose dependent manner (**a**). *PSMB5* mRNA expression was amplified by qRT-PCR in CMY and BTZ-resistant CMY cell line variants (CMY BR50, CMY BR100, CMY BR200 and the CMY BR-200). The *PSMB5* mRNA levels were increased significantly in the four CMY-BR variants in a dose-dependent manner. In the CMY BR-200 cells (cultured for 6 months in the absence of BTZ) *PSMB5* mRNA levels decreased, but were still significantly higher than those of control (**b**). Expression of β5 subunit of the 20S proteasome was measured in BTZ-resistant CMY cell line variants after treatment with different concentrations of BTZ for 12 h and compared to that of the CMY parent cell line (**c**). The inhibition of CT-like activity was measured after 6 h of treatment with different concentrations of BTZ in CMY and the BTZ-resistant CMY cell line variants. The CT-like activity inhibition in the BTZ-resistant cells was reduced. Data are presented as percentage of the CT-like activity relative to control (y-axis) against BTZ-treatment (x-axis) (**d**)

We next investigated the mechanism of BTZ resistance in the CMY cell line. The full-length β5 subunit *PSMB5* was amplified and sequenced from both CMY-BR cells and the parental CMY cell line (Additional file 1: Figure S1A). An A362C mutation in *PSMB5* exon 2 causing a change from glutamine to proline (Q62P) was detected (Additional file 1: Figure S1B). The mutation was located at the alpha helix, away from the BTZ binding site (Additional file 1: Figure S1C). To verify whether the Q62P mutation causes BTZ resistance, HEK293A cells were transfected with either full-length *PSMB5 WT*, *PSMB5 Q62P* or vector control, and underwent hygromycin selection. Stably transfected cells were then measured for viability after exposure to different concentrations of BTZ. HEK293A cells transfected with *PSMB5 Q62P* were significantly resistant to BTZ compared with HEK293A transfected with *PSMB5 WT* or vector control (*p < 0.00001*) (Fig. 4a).

### β5 proteasome subunit mRNA and protein overexpression is associated with BTZ resistance

To study whether BTZ resistance affected the expression or function of the PSMB5 subunit, we used qRT-PCR to study the *PSMB5* mRNA levels in CMY cells, as well as in the CMY-BR variants. We used CMY cells that were made BTZ resistant using different nanomolar concentrations of BTZ; CMY BR50, CMY BR100, and CMY BR200. Furthermore, CMY BR200 cells were sub-cultured for an additional 6 months after withdrawal of BTZ (CMY BR-200) to study whether the BTZ resistance will be maintained without continuous exposure to the drug. The *PSMB5* mRNA levels were increased significantly in the four CMY-BR variants in a dose-dependent manner, reaching 4-fold in CMY BR200 cells. However, when CMY BR200 cells were cultured for an additional 6 months in the absence of BTZ (CMY BR-200) *PSMB5* mRNA levels decreased, but were still significantly higher than those of control (*p < 0.0001*) (Fig. 4b). Similarly, the protein levels of the β5 proteasome subunit increased in all the CMY-BR variants compared to the parent CMY cells, and decreased after withdrawal of BTZ from the culture medium (CMY BR-200) (Fig. 4c). This indicated that the increase in expression of PSMB5 might be a mechanism to compensate for BTZ resistance caused by the Q62P mutation. We next investigated whether the Q62P mutation affected the CT-like activity. CMY and CMY-BR cells were treated with different concentrations of BTZ and the CT-like activity was measured. While 6 nM of BTZ inhibited 75% of the CT-like activity in the parent CMY cells, it inhibited only 50% of the CMY-BR variant. The inhibition of CT-like activity was dose-dependent in both cell lines. This indicated that the capacity of BTZ to inhibit the CT-like activity in CMY-BR cells is retained but compromised (Fig. 4d).

### BTZ-resistant CMY and CMK cells are cross-resistant to other proteasome inhibitors but are sensitive to DSF/Cu^2+^

**Fig. 5:**
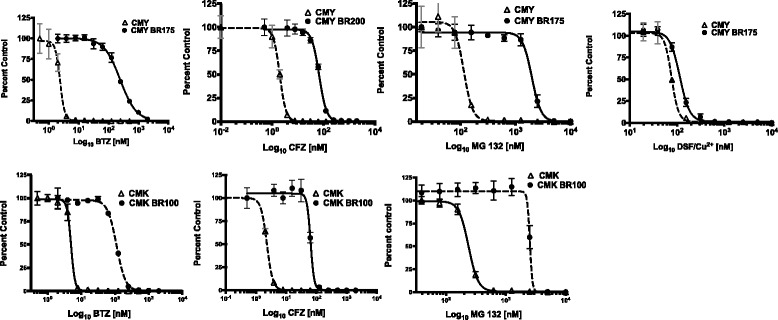
BTZ-resistant cell lines are cross-resistant to CFZ and MG-132 but sensitive to DSF/Cu^2+.^ CMY, CMY BR175, CMY BR200 and CMK BR100 were treated with several concentrations of BTZ, CFZ, MG 132 and DSF/Cu^2+^ for 72 h, and the dose response curves were plotted. All BTZ-resistant variants were cross-resistant to CFZ and MG-132 but sensitive to DSF/Cu^2+^

Since the CT-like activity in BTZ-resistant cells was maintained, we were interested to study whether other proteasome inhibitors would overcome BTZ resistance in the DS-AMKL cell lines CMY and CMK. The parent cell lines CMY and CMK, and their BTZ-resistant variants were treated with BTZ, CFZ, MG-132, or with DSF/Cu^2+^, and the IC_50_ values were determined (Table 2, Fig. 5). The IC_50_ values for BTZ ranged from 60 to 70 fold higher for CMY BR100 through CMY BR200, and 33 fold higher for CMK BR100 compared to their parent counterparts. All BTZ-resistant cell lines were also resistant to CFZ, with IC_50_ values ranging from 25 to 31 fold higher for CMY BR100 to BR200, and 17-fold higher for CMK BR100, compared to their parent counterparts, indicating that CFZ was not able to overcome BTZ resistance in this experimental setting. All BTZ-resistant variant cell lines were also resistant to MG132, but sensitive to DSF/Cu^2+^, with IC_50_ values similar to the parent cell lines (Table 2, Fig. 5). This suggested that DSF/Cu^2+^, although previously reported to have proteasome inhibitory properties, most likely acts through a different mechanism than BTZ, CFZ and MG-132. Moreover, these findings suggest that DSF/Cu^2+^ may be used to target AML cells that are resistant to BTZ or CFZ.

### DSF/Cu^2+^ inhibits colony formation similar to BTZ and Ara-C

We next compared the capacity of BTZ, Ara-C and DSF/Cu^2+^ to inhibit colony formation. THP-1 cells were pre-treated with several concentrations of DSF/Cu^2+^, BTZ and Ara-C for 72 h and then plated in Methocult. The number of colonies formed decreased with increasing doses of DSF/Cu^2+^, similar to both Ara-C and BTZ, indicating that all three drugs inhibited colony formation with the same capacity (Additional file 2: Figure S2A).

### DSF/Cu^2+^ induces apoptosis and proteasome inhibition independent of chymotrypsin-like activity

**Fig. 6:**
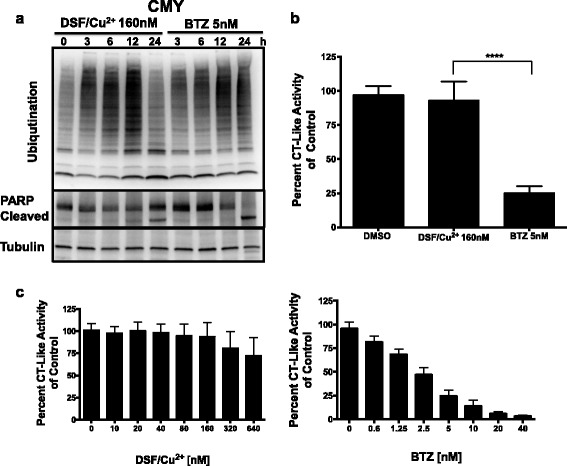
DSF/Cu^2+^ induces proteasome inhibition and apoptosis independent of the CT-like activity. Lysates from CMY cells treated with IC_90_ doses of DSF/Cu^2+^ (160 nM) or BTZ (5 nM) were prepared at the indicated time points. Western blot analysis was performed using an antibody against ubiquitin (**a**, *upper panel*), against PARP (**a**, *middle panel*), and Tubulin was used as a loading control (**a**, *lower panel*). CT-like activity of CMY cells treated with DSF/Cu^2+^ or BTZ at the concentrations described in (**a**) was measured at 12 h and plotted as a percent of control (y-axis) against drug treatment (x-axis). BTZ significantly inhibited the CT-like activity compared to DSF/Cu^2+^ and vehicle control (**b**). The same experiment was performed at 12 h using increasing doses of both BTZ and DSF/Cu^2+^ (**c**)

Although DSF/Cu^2+^ was previously reported to inhibit the proteasome, our findings suggest that it may act through a different mechanism than BTZ and CFZ (Fig. 5). We, therefore, studied the effect of DSF/Cu^2+^ on ubiquitination, and on CT-like activity of the 20S proteasome subunit, in comparison to BTZ. CMY cells were treated with the IC_90_ dose of both BTZ and DSF/Cu^2+^ for different time points. Cellular lysates were analyzed by western blot using an antibody against ubiquitin. DSF/Cu^2+^ induced ubiquitination similar to BTZ, which was most pronounced at 12 h (Fig. 6a, upper panel). However, using the same DSF/Cu^2+^ concentration at 12 h did not inhibit the CT-like activity, similar to BTZ, which significantly inhibited the CT-like activity in comparison to both vehicle control and DSF/Cu^2+^ (Fig. 6b). To confirm these findings, we treated the CMY cells with increasing concentrations of DSF/Cu^2+^ and BTZ. We observed a slight decrease in the CT-like activity only at very high doses (320 and 640 nM), while BTZ inhibited the CT-like activity in a dose-dependent manner from the 0.6 nM, as expected (Fig. 6c). This suggested that DSF/Cu^2+^ and BTZ may have different mechanisms of inhibiting the proteasome. Inhibition of protein degradation induces apoptosis; therefore, we next studied PARP cleavage by western blot. Both DSF/Cu^2+^ and BTZ induced PARP cleavage after 24 h using the same concentrations that induced ubiquitination at earlier time points (Fig. 6a, middle panel). In addition, DSF/Cu^2+^-induced apoptosis was confirmed by Annexin V/Propidium iodide staining and flow cytometry (Additional file 2: Figure S2B).

## Discussion

Pediatric patients with DS-AMKL are treated with the same chemotherapeutic agents as patients without DS. However, they experience severe therapy-related toxicities compared to the patients with non DS-AMKL, and the prognosis of the DS-AMKL patients with relapsed or refractory disease is very poor [30], underscoring the requirement for less toxic alternative therapeutic strategies for this subgroup of patients. In this study, we provide evidence that DSF/Cu^2^ is highly cytotoxic not only in cell lines developed from Down syndrome patients with relapsed/refractory AML, but also in 12 additional AML cell lines representing eight AML subtypes, and it overcomes Ara-C and BTZ resistance.

Ara-C is the key standard agent used in the treatment of AML. Pediatric patients with DS-AMKL are highly responsive to Ara-C in comparison to non DS-AMKL [31]. In the present study, this type of response was seen in the DS-AMKL cell line CMK cells compared to the non-DS-AMKL CMS cell line. However, the DS-AMKL cell line CMY was 25-fold more resistant to Ara-C compared to CMK cells, confirming previous reports [7]. Increased sensitivity to Ara-C was reported to be due to increased expression of the enzyme cystathionine-[β]-synthase (CBS) [32], and to the presence of somatic mutations in the transcription factor gene *GATA-1* in leukemia cells of patients with DS-AMKL [33]. The combination of trisomy 21 and the *GATA-1* mutations increases the amount of active Ara-C metabolites present in DS-AMKL cells, thereby enhancing its cytotoxicity [34]. However, the mechanisms of Ara-C resistance in the DS-AMKL CMY cells are not known. In this study, the presence of a high percentage of ALDH^bright^ cells in this cell line may contribute to its Ara-C resistance. ALDH is highly expressed in LSCs [35], which are thought to contribute to drug resistance and relapse in AML. Therefore, in order to prevent disease recurrence, this subpopulation of stem cells must be eliminated. BTZ was previously shown to induce apoptosis in the “stem cell like” blasts from AML patients [9], so it was used in the present study to target the ALDH-positive “stem-like” cells in the Ara-C resistant CMY cell line, in comparison to the ALDH inhibitor DSF/Cu^2+^. While approximately 1% of the ALDH^bright^ cell population of CMY cells remained resistant to BTZ, they were very sensitive to DSF/Cu^2+^, indicating that DSF/Cu^2+^, but not BTZ, is equally cytotoxic to both the AML proliferating cells, as well as to the ALDH^bright^ “stem-like” cells. This is in agreement with previous studies demonstrating that DSF targets ALDH-positive cancer stem cells in breast cancer [36], in glioblastoma [29, 37], and in Non-Small Cell Lung Cancer (NSCLC) [38].

To further understand the mechanism of BTZ resistance in the DS-AMKL cell lines, we generated BTZ-resistant variants of both CMY and CMK cell lines and detected a novel A362C mutation in the *PSMB5* exon 2 in the CMY-BR cells, causing a change from glutamine to proline (Q62P). We confirmed that this mutation caused BTZ resistance in transformed HEK293A cells. However, this mutation did not completely abrogate the CT-like proteasome activity, and was accompanied by an upregulation of the β5 subunit on the mRNA and protein levels. This is consistent with previous studies showing that cells with acquired BTZ resistance have upregulated mutant β5 subunit, which serves as a compensatory mechanism to retain sufficient CT-like proteasome activity [39]. To find compounds that are clinically used in the treatment of DS-AMKL and would overcome BTZ resistance in this model, we tested the second-generation proteasome inhibitor CFZ, Ara-C, VP-16, daunorubicin, in comparison to MG 132 and DSF/Cu^2+^. Interestingly, the CMY-BR cells became more resistant to Ara-C at higher concentrations. For Ara-C to function as an anti-tumor agent, it is sequentially phosphorylated, eventually to Ara-C triphosphate (Ara-CTP), which is incorporated into DNA strands during the S phase of the cell cycle, thereby inhibiting DNA synthesis and causing S-phase arrest [40]. In the present study, Ara-C induced S-phase arrest in the parent CMY cell line, confirming previous studies in AML cells [9]. However, in the CMY-BR variant, only G1 arrest was observed. BTZ was previously reported to decrease the levels of CCND1, CDK4 and CDK2 (required for G1/S transition), while it increases those of p27 and p21 (inhibitors of CDK2) [9, 41]. Cells would subsequently accumulate in G1, as observed in the present study. G1 arrest, here, may protect the cells from Ara-C effects, thus supporting previous findings using MCL cell lines, in which G1 arrest by abemacilib (a CDK4/6 inhibitor) protected the cells from Ara-C damage [42]. Our results may also explain the failure of combined BTZ and Ara-C therapy to show substantial improvement in the OS in pediatric patients with relapsed/refractory or secondary AML [43]. Our results show that the BTZ resistant variants were cross-resistant to CFZ and MG 132 but sensitive to DSF/Cu^2+^, which suggests that DSF/Cu^2+^ interferes with the proteasome through a different mechanism than CFZ and BTZ. DSF/Cu^2+^ induced ubiquitination, apoptosis and PARP cleavage similar to BTZ, but it did not inhibit the CT-like activity. This is in contrast to previous studies using breast cancer cell lines [27] and cultured glioma stem cells [37] that showed that DSF/Cu^2+^, and Cu^2+^ alone, but not DSF, caused inhibition of CT-like activity, suggesting that the proteasome effect of DSF/Cu^2+^ is due to copper [44]. Our results support the notion that unlike BTZ, DSF/Cu^2+^ complexes target the 26S proteasome rather than the 20S [45]. A likely target could be the JAB1/MPN/Mov34 metalloenzyme (JAMM) domain of the POH1 subunit within the lid of the 19S proteasome, as was proposed in [46]. POH1, a member of the JAMM domain deubiquitinases, is necessary for activity of the 26S proteasome [47] and for cell viability [48]. Inhibition of the 26S proteasome-associated deubiquitinases result in apoptosis and in vivo inhibition of tumor progression [49]. Furthermore, proteasome inhibition leads to the accumulation of misfolded proteins and possible toxic protein aggregates, which typically induces the unfolded protein response and heat shock protein activation [50]. Other reported mechanisms of DSF/Cu^2+^ include the induction of reactive oxygen species, leading to pro-apoptotic JNK activation [51], as well as the inhibition of the HER2 pathway in breast cancer [36].

## Conclusions

Despite the clinical successes of Ara-C and BTZ, inherent and acquired resistance to these drugs remains a clinically significant problem, and a major challenge for patients with AML. Overcoming resistance to Ara-C and BTZ would offer a new treatment option for these patients. In this study, we provide evidence that the FDA-approved, well-tolerated, inexpensive, oral drug disulfiram, in combination with clinically relevant copper concentrations, can overcome both Ara-C and BTZ resistance in cell lines from patients with DS-AMKL. We also identify a novel mutation underlying acquired BTZ resistance that may be used as a biomarker to identify BTZ-resistant patients, who may not benefit from subsequent or combined treatment with CFZ or Ara-C, but may be responsive to DSF/Cu^2+^. Collectively, results from the present study support the clinical development of DSF/Cu^2+^ as a less toxic and efficacious treatment approach to be tested in patients with relapsed/refractory DS-AMKL with poor outcome.

## Additional files

Additional file 1: Figure S1. Structure of the *PSMB5* gene and mRNA (A). DNA chromatogram showing the location of *PSMB5* Q62P mutation in exon 2 (B). 3D protein structure of the *PSMB5* Q62P performed using Swiss Prot database, showing the location of the *PSMB5* Q62P on the alpha helix (blue arrow). A previously reported *PSMB5* mutation (Cys63) is shown (C). (PPTX 419 kb)
Additional file 2: Figure S2. DSF/Cu^2+^ inhibits colony formation and induces apoptosis in AML cell lines. THP-1 cells were treated with several doses of either DSF/Cu^2+^, BTZ or Ara-C. Viability was measured at 72 h by Trypan Blue dye exclusion assay. Viable cells were seeded in Methocult and colonies counted after 10 days. Viability and number of colonies are presented as percent of untreated control (A). CMY cells were exposed to increasing concentrations of DSF/Cu^2+^ for 24 h and apoptosis was assessed using Annexin V (x-axis) and propidium idodide (PI) (y-axis). Results are represented in four quadrants. For all treatment options, the bottom left quadrant represents live cells [Annexin V(−)/propidium iodide (PI) (−)], the bottom right quadrant represents the percentage of cells in early apoptosis [Annexin V(+)/PI (−)], the upper right quadrant represents the percentage of cells in late apoptosis [Annexin V (+)/PI (+)] and the upper left quadrant represents the percentage of cells in necrosis [Annexin V (−)/PI (+)]. (PPTX 223 kb)

## Abbreviations

ALDH: Aldehyde dehydrogenase
AML: Acute Myeloid Leukemia
Ara-C: Cytarabine
BTZ: Bortezomib
CFZ: Carfilzomib
CTG: Cell Titer Glo
DDR: Drug dose response
DEAB: N, N-diethylaminobenzaldehyde
DMSO: Dimethyl sulfoxide
DS: Down syndrome
DS-AMKL: Down syndrome-associated AML
DSF: Disulfiram
LSC: Leukemia stem cells
VP-16: Etoposide
