# Supplementary material for: Disulfiram overcomes bortezomib and cytarabine resistance in Down-syndrome-associated acute myeloid leukemia cells
Source: J Exp Clin Cancer Res. 2017 Feb 1;36:22. doi: 10.1186/s13046-017-0493-5 (PMC5286849; doi:10.1186/s13046-017-0493-5)
Supplement: Additional file 1: Figure S1. — Structure of the PSMB5 gene and mRNA (A). DNA chromatogram showing the location of PSMB5 Q62P mutation in exon 2 (B). 3D protein structure of the PSMB5 Q62P performed using Swiss Prot database, showing the location of the PSMB5 Q62P on the alpha helix (blue arrow). A previously reported PSMB5 mutation (Cys63) is shown (C). (PPTX 419 kb) [file 13046_2017_493_MOESM1_ESM.pptx]

## Slide 1
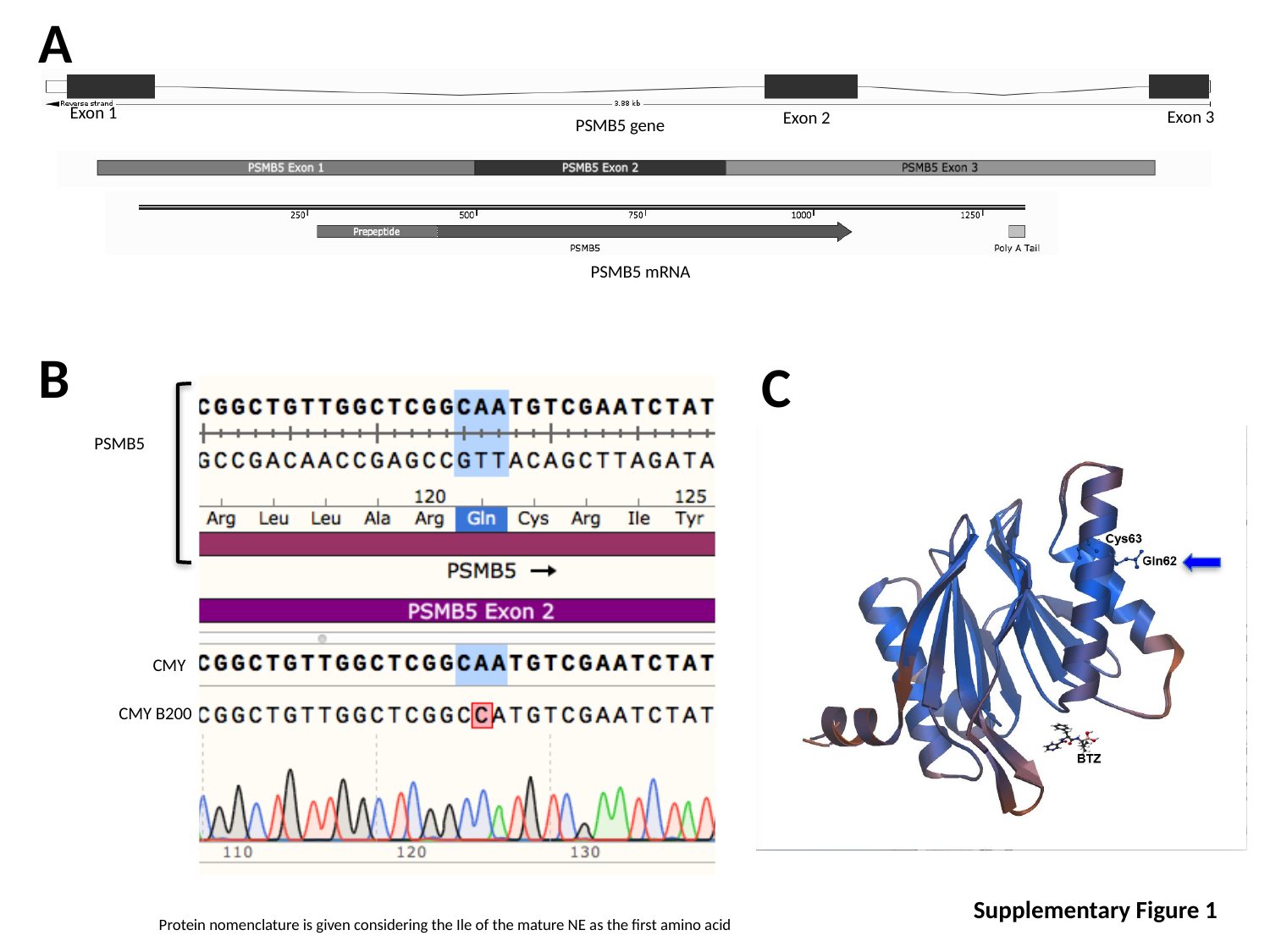

A
Exon 1
Exon 3
Exon 2
PSMB5 gene
PSMB5 mRNA
B
C
PSMB5
CMY
CMY B200
Protein nomenclature is given considering the Ile of the mature NE as the first amino acid
Supplementary Figure 1
