# Supplementary figures and images for: Disulfiram overcomes bortezomib and cytarabine resistance in Down-syndrome-associated acute myeloid leukemia cells
Source: J Exp Clin Cancer Res. 2017 Feb 1;36:22. doi: 10.1186/s13046-017-0493-5 (PMC5286849; doi:10.1186/s13046-017-0493-5)

## Slide 1
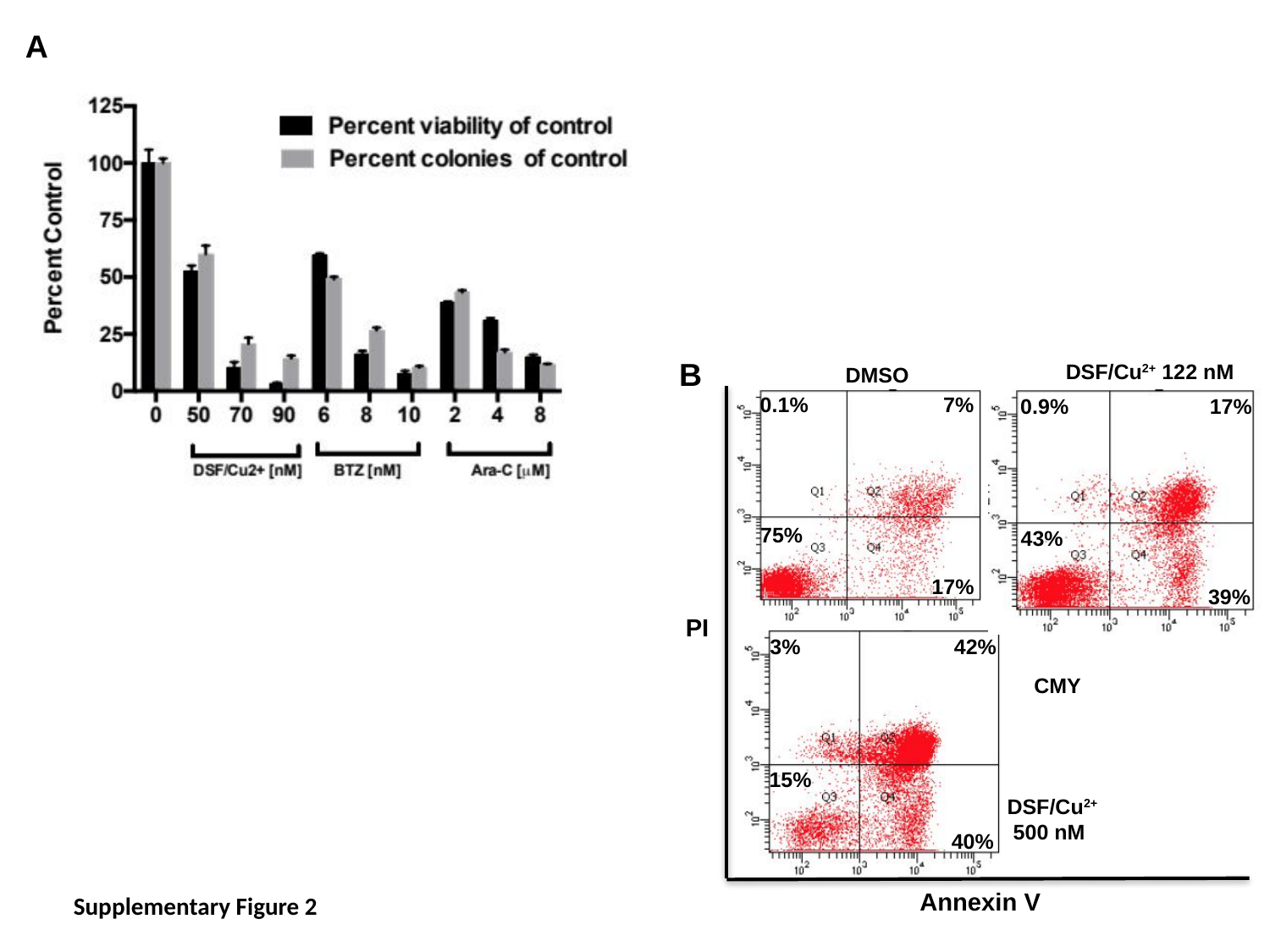

A
B
DSF/Cu2+ 122 nM
DMSO
0.1%
7%
75%
17%
17%
0.9%
43%
39%
PI
3%
42%
15%
40%
CMY
DSF/Cu2+
 500 nM
Annexin V
Supplementary Figure 2

Supplement: Additional file 2: Figure S2. — DSF/Cu2+ inhibits colony formation and induces apoptosis in AML cell lines. THP-1 cells were treated with several doses of either DSF/Cu2+, BTZ or Ara-C. Viability was measured at 72 h by Trypan Blue dye exclusion assay. Viable cells were seeded in Methocult and colonies counted after 10 days. Viability and number of colonies are presented as percent of untreated control (A). CMY cells were exposed to increasing concentrations of DSF/Cu2+ for 24 h and apoptosis was assessed using Annexin V (x-axis) and propidium idodide (PI) (y-axis). Results are represented in four quadrants. For all treatment options, the bottom left quadrant represents live cells [Annexin V(−)/propidium iodide (PI) (−)], the bottom right quadrant represents the percentage of cells in early apoptosis [Annexin V(+)/PI (−)], the upper right quadrant represents the percentage of cells in late apoptosis [Annexin V (+)/PI (+)] and the upper left quadrant represents the percentage of cells in necrosis [Annexin V (−)/PI (+)]. (PPTX 223 kb) [file 13046_2017_493_MOESM2_ESM.pptx]
